# Supplementary material for: Bulk Segregant Analysis Sequencing and RNA-Seq Analyses Reveal Candidate Genes Associated with Sepal Color Phenotype of Eggplant (Solanum melongena L.)
Source: Plants (Basel). 2024 May 16;13(10):1385. doi: 10.3390/plants13101385 (PMC11124939; doi:10.3390/plants13101385)
Supplement: Supplementary file 1 [file plants-13-01385-s001.zip › Supplementary Materials.pdf]

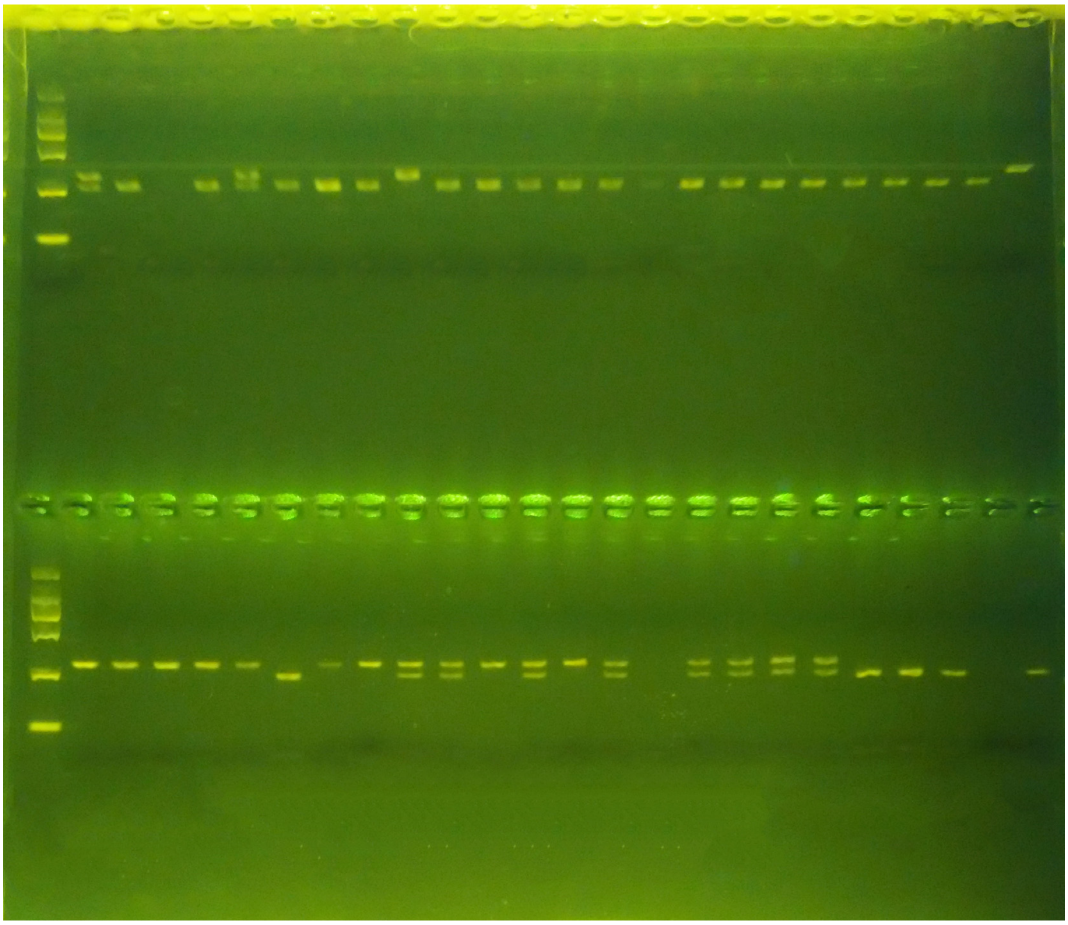

Supplementary Figure1 Electropherogram of CAPS molecular labeling.

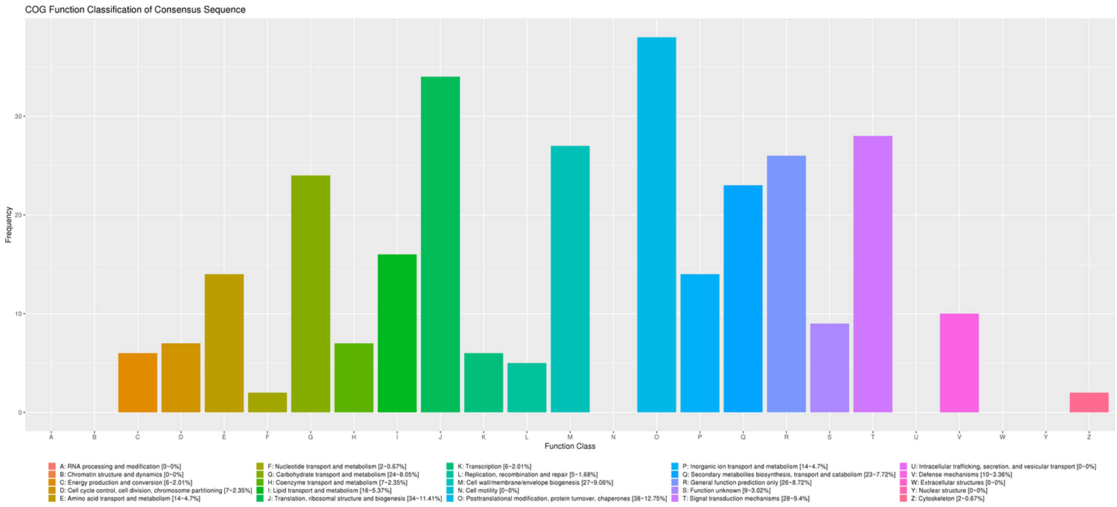

Supplementary Figure 2 Statistical results of COG classification of genes in the associated region.

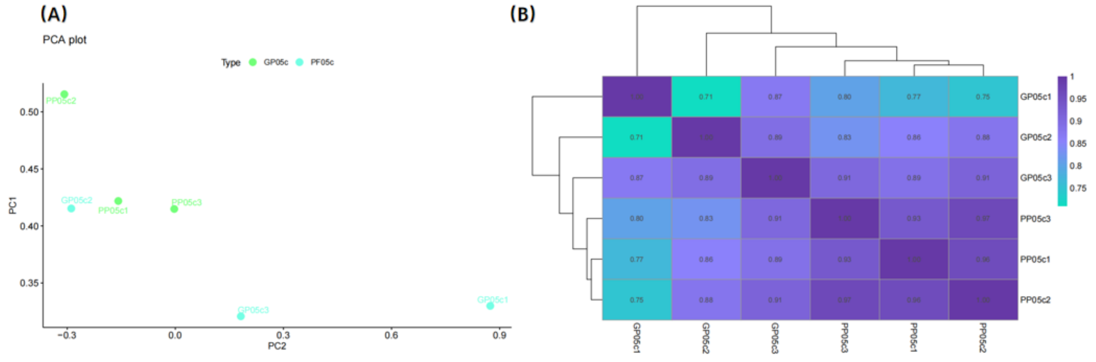

Supplementary Figure 3 A PCA plot of mRNA principal component analysis, B Pearson correlation coefficient among counts of transcriptome data. Numbers 1, 2 and 3 behind the GP05 andPP05 represent the biological replicates.

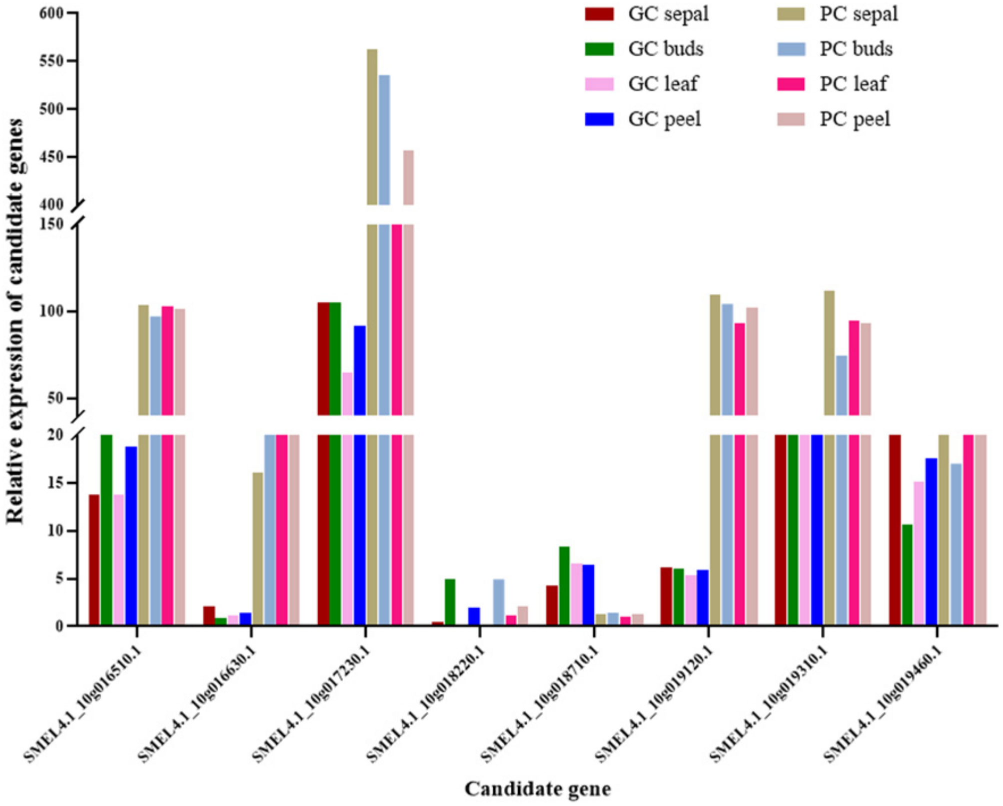

Supplementary Figure 4 The expression of candidate genes in different tissues of purple sepals (PC) and green sepals (GC) was detected using qRT-PCR.
